# Battle Creek, Michigan

**Published:** 1890-08

**Authors:** 


BATTLE CREEK, MICH., JUNE 24TH, 1890.

EDITOR OF DENTAL REGISTER:

*Dear Sir*:—The contribution of my (almost) octogenarian friend "Uncle Jerry" Robinson, D.D.S., to the June number of the "DENTAL REGISTER" revives some early recollections of dentists and the dental art as known and practiced a half century ago which may have some interest perhaps to some of the younger generation of "Tooth Carpenters," as the "better half" of my friend Dr. M., oftentimes facetiously speaks of them to him. The first experimental knowledge I had of a dentist was when I was a boy about fifteen years old, in the year 1835 or 1836, in the little rural village of Cummington, Mass., the birthplace of William Cullen Bryant, United States Senator Henry S. Dawes, Gideon Lee, (one of New York's ex-Mayors), Fordyce Knapp, of New York, and many others of national reputation and fame. Dr. Bigelow, of New York City, occasionally "summered" there, and he impressed me with profound awe and admiration dressed in his full suit of white linen. One day suffering with tooth ache I "ushered myself" timidly into his presence and "quaked in my boots," or more likely in my bare feet, as he sat me down and resting my head against his knee proceeded to punch some sharp instrument into my sensitive molars. The result was his filling two or three teeth which heretofore had been quiet and uncomplaining and made some application to the aching member, quieting for the time, so that he afterwards "plugged" it, but in a few days after was obliged to extract it; which experience filled me with a holy horror of dentists and their "probangs" for many years afterwards. My next experience with a dentist was some two or three years afterwards in Northampton, Mass. I was engaged teaching a country school in the little town of East Hampton, Mass., when I was seized with a raging tooth ache while at supper one evening, and I faced a severe northwester a couple of miles, where at Capt. Levi Burt's, I knew there was a superannuated old doctor, lately returned from Ohio, who sometimes "pulled teeth." I remember his seating me on a stool in front of the big fire place to get better light, and taking an old handkerchief proceeded to wrap it around the fulcrum of an old-fashioned turnkey, and with my head between his knees applied the *twist*, which resulted in cracking "square off" two rampant "bicuspids" leaving me in a far worse plight than he found me. After a sleepless night, the next morning a kind friend drove me in a sleigh to Northampton, where Dr. Walker, a celebrated dentist relieved my sufferings—my first experience with a pair of forceps. Some tin foil fillings of his did me good service for many years. In the winter of 1840–1, I called at the office of a Dr. Bradley, in Philadelphia, having some dental work done, and was much interested in seeing him carving an upper set of teeth from ivory. He was a young man and I think it was from him that I took the "inspiration" which resulted in a life-long devotion to the practice of dentistry, notwithstanding the unpleasant experiences I had had as a patient. My next experience and acquaintance was with Dr. Dana, of Darlington, South Carolina, at Sumter and Bradford Springs, S. C., who executed some fine gold work for me and whose fillings remained with me for many years, and to whose instruments and books I afterwards succeeded, upon his death in 1843. Afterwards in the office of Dr. Willard Clough, in Pittsfield, Mass., I had opportunity to further pursue the studies in medicine and dental surgery, the former of which I had begun with Dr. I. M. Hume, at Wayne, Mich., and continued with Dr. Thomas M. Dick, in Sumter, S. C., finally locating in Battle Creek, Mich., in the year 1845, where for forty-five years I have practiced dentistry, and medicine during a few of the first years of my sojourn here. In my early experience no teeth were furnished in sets, but we had to select and match them up from promiscuous teeth, and as nothing was known then of the atmospheric pressure principle, we depended on spiral springs attached to the rear end of upper and lower plates to keep them in place. "Putman's Central Cavity Plate" was among the first attempts at retaining plates by suction, though I remember my old preceptor, Dr. Willard Clough, anticipated it somewhat by making the upper plates "double" and punching holes through the upper one thereby securing considerable "suction." I have some samples of the first "mineral teeth" as they were called, made in Paris, and I remember the first attempts in the manufacture of "gum teeth" (single) when the color just tipped the top of the tooth—afterwards resulting in blocks of two and three, with a wide gum color above the tooth. There were no dental depots in those days and a Mr. Barrett, of Albany, N. Y., was the only gold beater I knew. He also supplied us with teeth and forceps. Chevalier of New York was the principal manufacturer of dental instruments especially forceps, though the oldest I have, have the name "Rose, New York" stamped upon them. Having seen dentistry as an art "evolve" from such humble origin it is with much pride that I behold it at the present day asserting itself as a science and contributing in a large degree to the comfort and civilization of the 19th century.—C. E. Bartlett, M.D.

To the Register:—

Reading an article of Dr. J. A. Robinson on "Evolution in Dentistry" taken from the Register in the June number of the *Western Journal*, I see that the doctor speaks of the use of sand paper strips as far back as the days of Harwood, Tucker, Bemis, and Flagg, of Boston, Mass., so I imagine that its use originated in Yankeedom. How it was introduced into New York I will tell. In 1862, when Dr. Atkinson opened his first office at 18 West 11th street, he was making a clinic on one of my teeth with an exposure of pulp. It was the first time I had seen a filling condensed with the mallet; no engine, no rubber dam. The doctor dipped his first pledget of gold in creosote, which gave a taste for months afterwards, but the tooth and filling are doing good service today and never has there been any disturbance of the pulp. There were present at the clinique Drs. Wm. H. Allen and W. W. Sheffield, of New London, Conn., and S. P. Miller, of Worcester, Mass. While Dr. Atkinson was trimming down the filling as was the custom with a file, Dr. Miller said "Doctor, why don't you use sand paper? It is very useful." A, once paper was procured and strips were cut and applied, and we all acknowledged its utility. From that day its use commenced and was at once spoken of in society as one of the useful adjuncts of practice. And in all these years how we have evoluted.

Geo. A. Mills, New York City.

---

Richmond, Va., July 11th, 1890.

The Virginia State Dental Association will hold its 21st annual session in the High School building at Roanoke, Virginia, Tuesday, August 26, 1890, begining at 9 o'clock A.M.

We confidently expect this to be the largest and best meeting the Association has ever held. All members of the profession are invited to attend and will receive a cordial welcome.

J. Hall Moore, Corresponding Secretary.

The State Board of Dental Examiners will meet at the same time and place for the examination of candidates to practice dentistry in the State. All applicants must be graduates of some reputable dental college. W. E. Norris, Secretary.

The annual meeting of the Post Graduate Dental Association of the United States was held at Chicago, June 25, 1890, and the following gentlemen were elected officers:

Pres., Geo. H. Cushing, M.D., D.D.S., Chicago, Ill; Vice-Pres., Dr. R. H. Cool, Oakland, Cal.; Sect'y and Treas., Lewis S. Tenney, D.D.S., Chicago, Ill; Executive Committee—R. B. Tuller, D.D.S., Chicago, Ill., Dr. J. M. Gallohugh, Chenoa, Ill., Dr. G. W. Milton, Silvertown, Colo.

This association is but a year old, but it starts out with good prospects of becoming a large and popular national organization, and has a grand work before it. Its object, aside from the same general one of most Dental Societies, is to particularly encourage and stimulate post graduate studies and the establishment of facilities for the same in dental colleges. It also contemplates, when its membership will admit of it, establishing a systematic course of home study with benefits not unlike the Chatauqua Literary Society perhaps, but the plan is not yet sufficiently developed to admit of outlining at this time.

While the name "Post Graduate" would imply an association of graduates only, the broad view is adopted of extending the work among all legal practitioners who may desire to join and co-operate, but practitioners not graduates are not eligible to membership until they have passed a post graduate or practitioners course in some reputable and recognized dental college.

Members of the profession who desire to become members of the Post Graduate Association should correspond with the secretary, Dr. Lewis S. Tenney, 96 State St., Chicago. The membership fee is $1. Annual dues, payable in advance, $1. Certificates of membership are issued when the member duly qualifies. Membership may be obtained through correspondence when evidence of eligibility is presented.

---

Columbus, Ohio, July 10, 1890.

Editor Register:—By inserting the following in the Register, until the time for meeting, you will confer a favor upon the committee.

To the Members of the Dental Profession. Your attention is called to the next meeting of the Ohio State Dental Society, to be held at Columbus, October 28, 29, and 30, 1890. You are cordially invited to be present and participate in the meetings if possible. Those having new appliances or methods of practice for the good of the profession are especially solicited to communicate with the committee who will take pains in offering an opportunity for the proper presentation of same.

A. F. Emminger,
W. H. Todd,
Otto Arnold, Ex. Com.

